# Identification of SARS-CoV-2 variants using viral sequencing for the Centers for Disease Control and Prevention genomic surveillance program

**DOI:** 10.1186/s12879-022-07374-7

**Published:** 2022-04-25

**Authors:** Chirayu Goswami, Michael Sheldon, Christian Bixby, Mehdi Keddache, Alexander Bogdanowicz, Yihe Wang, Jonathan Schultz, Jessica McDevitt, James LaPorta, Elaine Kwon, Steven Buyske, Dana Garbolino, Glenys Biloholowski, Alex Pastuszak, Mary Storella, Amit Bhalla, Florence Charlier-Rodriguez, Russ Hager, Robin Grimwood, Shareef A. Nahas

**Affiliations:** 1 Infinity-Biologix LLC, 30 Knightsbridge Road, Piscataway, NJ 08854 USA; 2 Illumina, 5200 Illumina Way, San Diego, CA 92122 USA; 3 Rutgers University, 559 Hill Center, 110 Frelinghuysen Rd, Piscataway, NJ 08854 USA; 4 Vault Health, 115 Broadway Suite 1800, 18th Floor, Dobbs Ferry, NY 10522 USA

**Keywords:** COVID-19, SARS-CoV-2, Centers for Disease Control, Next generation sequencing, Reverse transcription polymerase chain reaction, Cycle threshold, Lineage, Variant

## Abstract

**Background:**

The Centers for Disease Control and Prevention contracted with laboratories to sequence the SARS-CoV-2 genome from positive samples across the United States to enable public health officials to investigate the impact of variants on disease severity as well as the effectiveness of vaccines and treatment. Herein we present the initial results correlating RT-PCR quality control metrics with sample collection and sequencing methods from full SARS-CoV-2 viral genomic sequencing of 24,441 positive patient samples between April and June 2021.

**Methods:**

RT-PCR confirmed (N Gene Ct value < 30) positive patient samples, with nucleic acid extracted from saliva, nasopharyngeal and oropharyngeal swabs were selected for viral whole genome SARS-CoV-2 sequencing. Sequencing was performed using Illumina COVIDSeq™ protocol on either the NextSeq550 or NovaSeq6000 systems. Informatic variant calling, and lineage analysis were performed using DRAGEN COVID Lineage applications on Illumina’s Basespace cloud analytical system. All sequence data and variant calls were uploaded to NCBI and GISAID.

**Results:**

An association was observed between higher sequencing coverage, quality, and samples with a lower Ct value, with < 27 being optimal, across both sequencing platforms and sample collection methods. Both nasopharyngeal swabs and saliva samples were found to be optimal samples of choice for SARS-CoV-2 surveillance sequencing studies, both in terms of strain identification and sequencing depth of coverage, with NovaSeq 6000 providing higher coverage than the NextSeq 550. The most frequent variants identified were the B.1.617.2 Delta (India) and P.1 Gamma (Brazil) variants in the samples sequenced between April 2021 and June 2021. At the time of submission, the most common variant > 99% of positives sequenced was Omicron.

**Conclusion:**

These initial analyses highlight the importance of sequencing platform, sample collection methods, and RT-PCR Ct values in guiding surveillance efforts. These surveillance studies evaluating genetic changes of SARS-CoV-2 have been identified as critical by the CDC that can affect many aspects of public health including transmission, disease severity, diagnostics, therapeutics, and vaccines.

**Supplementary Information:**

The online version contains supplementary material available at 10.1186/s12879-022-07374-7.

## Background

As the number of patients confirmed to be infected with the severe acute respiratory syndrome coronavirus 2 (SARS-CoV-2) virus and developing coronavirus disease 2019 (COVID-19) increased, international sequencing efforts began to determine genetic variations in SARS-CoV-2 genes that may potentially increase viral transmission and pathogenicity [1]. However, the number of genomes sequenced within the United States (U.S.) deposited in the online genome repository, GISAID, in March of 2021 represented only 1.6% of the number of COVID-19 cases that month. As international sequencing efforts increased, variants of high concern were identified in the United Kingdom (SARS-CoV-2 B.1.1.7), and in the U.S. (B.1.1.7, B.1.351, P.1, B.1.427, and B.1.429). Among the SARS-CoV-2 variants of concern, Beta B.1.351 (South Africa) and Gamma P.1 (Brazil) have high potential to reduce the efficacy of some vaccines [2–5]. The B.1.617 variant (India) has become a variant of interest for its high transmission rate and ability to evade immune responses [6]. At the time of submission of this manuscript the first cases of the South African variant B.1.1.529 (Omicron) have been detected, but not yet identified in the U.S. [21] These genomic sequencing efforts have allowed scientists to identify not only SARS-CoV-2 positive patients and viral sequence variants, but to also monitor how new viral variants evolve and to understand how these changes affect the characteristics of the virus, with this information ultimately being used to better understand health impacts. In addition, these variants of concern are actively being monitored to determine if they may reduce the efficacy of currently approved vaccines against SARS-CoV-2 [7].

**Fig. 1:**
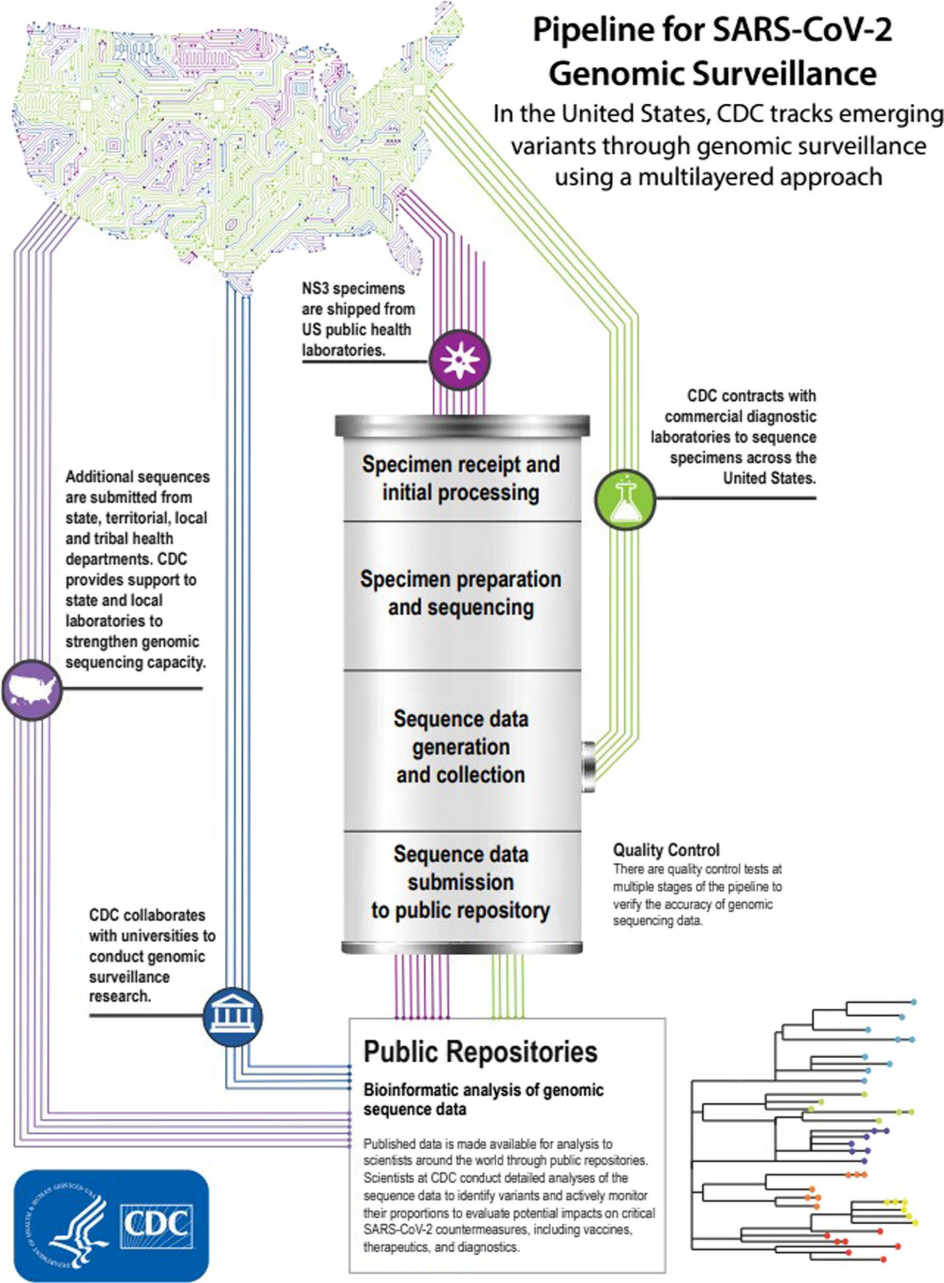
CDC tracking of emerging variants through the pipeline for genomic surveillance (https://www.cdc.gov/coronavirus/2019-ncov/variants/cdc-role-surveillance.html). As part of the CDC National SARS-CoV-2 Strain Surveillance (NS3) System, contracted laboratories select for sequencing a set of deidentified specimens that were previously subjected to SARS-CoV-2 RT-PCR testing and determined to be positive. Generally, the samples then undergo a three-step process for generating sequence data. Specimen preparation and sequencing: SARS-CoV-2 RNA is extracted and converted to complimentary DNA, enriched, and loaded into the next-generation sequencers. Sequence reads are aligned to SARS-COV-2 reference strain using the k-mer detection method. Aligned reads are then used to generate the consensus sequence, call variants and lineage determination. The information along with sequencing quality control statistics are transferred to the CDC repository electronically. Published data are made available to scientists around the world through public repositories

In March of 2021, the Centers for Disease Control (CDC) contracted with large commercial diagnostic labs to sequence patient samples across the U.S. [8]. These laboratories committed to sequencing up to 6000 samples per week, with the capacity to scale up in response to the nation’s needs (Fig. 1). The purpose of this program is to perform routine analysis of genetic sequence data to enable the CDC and its public health partners to identify and characterize variant viruses—either new ones identified in the U.S. or those already identified abroad—and to investigate how variants impact COVID-19 disease severity and the effectiveness of vaccines and treatment. Infinity Biologix LLC (IBX), located in Piscataway NJ, was awarded one of these contracts to participate in this surveillance program.

In this study we describe the results of full-length genomic sequencing and surveillance of the SARS-CoV-2 virus from the first 24,441 confirmed positive saliva (SA) samples and a small subset of nasopharyngeal and oropharyngeal (NP, OP) swab-based samples sequenced between April and June 2021.

## Methods

### CDC laboratory surveillance program selection

IBX was designated by the CDC to participate in its Genomic Surveillance for SARS-CoV-2 Variants program [1]. This program conducts genomic surveillance using a random sampling of previously confirmed positive samples from across the U.S., including all 50 states, Washington DC, Puerto Rico and major U.S. territories and possessions. A target of up to 6000 genomic sequences per week was established including State and Zip Code as the required demographics data. CDC requested, when possible and if available, additional demographic data as age, sex, and ethnicity of the patients.

### Patients and sample acquisition

Diagnostic patient samples for SARS-CoV-2 testing arrived via multiple external test clients, approved by IBX and Food and Drug Administration (FDA). Demographic, vaccination, ethnicity, sex and age were available if provided by the patient during sample collection. Most samples (> 95%) were saliva-based and collected in Minnesota (MN) and New Jersey (NJ) given the presence of IBX laboratories in each state.

### Criteria for SARS-CoV-2 viral sequencing

The criteria for positive samples selection were established using a nucleocapsid protein (N Gene) Cycle Threshold (Ct) value threshold of ≤ 30.0 on the IBX TaqPath SARS-CoV-2 QPCR Assay. Per the CDC requirement, samples to be sequenced must have been no more than 10 days old following confirmation of a positive test result.

### RNA isolation

All samples selected for sequencing had nucleic acid freshly extracted from the primary sample source independently of the material extracted for the initial RT-PCR testing. Automated RNA extraction from either SA, NP or OP was carried out using the Chemagic Viral DNA/RNA 300 Kit H96 (CMG-1033-S, PerkinElmer) on the Chemagic 360 Nucleic Acid Extractor (2024-0020, Perkin Elmer).

### Real-time PCR for SARS-CoV-2

All samples were tested using the Infinity BiologiX TaqPath SARS-CoV-2 QPCR Assay according to the procedure detailed in FDA Emergency Use Authorization (EUA) #200090 to provide diagnostic results for the presence of SARS-CoV-2. This assay is approved for SA, OP and NP swab types [9]. Briefly, this assay quantitates expression of three viral loci, the N, Coronavirus Spike Protein (S) and Open Reading Frames (ORF1) ab genes, with the N gene Ct value being germane to the selection of samples to be sequenced in this study. Samples testing positive for the presence of SARS-CoV-2 were isolated and stored at 4 °C within 72 h after collection for potential further use. Samples were transferred to − 80 °C for permanent storage.

### Library preparation and sequencing

Sequencing libraries (500 initial samples tested) were prepared manually using the Illumina COVIDSeq Test protocol following the manufacturer’s guidelines (Illumina Inc., San Diego, CA) [10, 11]. All remaining libraries were then prepared in 96-well PCR plates (2 plates per run) on Sciclone® G3 NGSx Workstations (Perkin Elmer Inc, Waltham, MA). Following cDNA synthesis and amplification, PCR amplicons were tagmented (fragmented and tagged with adaptors) using IDT® for Illumina PCR Unique Dual Indexes Set 1–4 (384 Indexes). Libraries were purified and pooled following the manufacture’s guidelines (Illumina Inc., San Diego, CA). Library pools (each containing 94 samples, 1 CPC HT, 1 NTC) were quantified and analyzed for fragment size on a Tapestation 4200 (Agilent Technologies, Inc., Santa Clara, CA). Pools (96 samples) were then normalized to 4 nM and combined into a sequencing pool for a total of 384 samples (index sets 1–4). Sequencing pools were quantified on a Qubit 2.0 fluorometer (Invitrogen Inc., Waltham, MA) to ensure a 4 nM starting concentration for sequencing preparations on a NovaSeq 6000 and NextSeq 550 sequencing platforms. Sequence pools destined for the NovaSeq 6000 loaded onto an S4 flowcell (each lane loaded with 1 sequencing pool of 384 samples) following the manufacturer’s protocol for the Xp-loading workflow (Illumina Inc., San Diego, CA). For sequencing performed on the NextSeq 550 platforms, 1 sequencing pool (384 samples) loaded onto a High Output flowcell following the manufacturer’s protocol (Illumina Inc., San Diego, CA). Across all sequencing platforms parameters were as follows: 2 × 75 bp paired-end, dual-indexed reads. Approximately 5000 samples were run on the NovaSeq 6000 and 15,000 on the NextSeq 550.

### Data analysis

FASTQ files were generated for each sample after demultiplexing of the raw sequencing data on Base Space Sequence Hub (BSSH). Detection of the virus, generation of the consensus sequence and lineage/clade determination was performed using the DRAGEN COVID Lineage App v3.5.1 and v3.5.2 on BSSH.

### Statistical analysis

Spearman’s rank correlation coefficient, a rank-based analogue of Pearson’s correlation, was used to measure the possibly non-linear but still monotonic relationship between mean Ct and % Genome Coverage. Tukey’s test was used to test for differences in mean Ct value among the three collection methods.

### Viral detection

Each sample was evaluated for the presence of the virus in the sequencing data using a k-mer based algorithm prior to performing variant calling steps. Each read was broken down into all possible contiguous 32 bases segments (32-mers) and compared to a pre generated list of 32-mers from all the amplicons in the CovidSeq assay (98 from SARS-CoV-2 and 11 from human control genes). An amplicon was considered detected when at least 150 k-mers were matched between the sample and reference. When 5 or more SARS-CoV-2 amplicons were detected, the virus was recorded as present in the sample and the variant calling pipeline was invoked.

### Alignment, variant calling and consensus

Reads were aligned against the NC_045512.2 sequence (Severe acute respiratory syndrome coronavirus 2 isolate Wuhan-Hu-1) using the DRAGEN Map/Align algorithm. Reads pairs that aligned to the same start and end positions were marked as duplicate by default (only the highest quality pair is retained for variant calling). No quality pre-filtering of the data was performed by default, but portions of reads that do not match the reference were soft clipped by the aligner (removed for variant calling but retained in the Binary Alignment Map (BAM) file) to eliminate mismatches due to a drop in read quality or chimeric artifacts from the PCR. For variant calling, the DRAGEN Somatic default down sampling parameters were used (no more than 10,000 reads per 300 base window around a variable position and no more than 50 reads starting at the same position were considered, random removal with fixed seed for reproducibility). Each variant was then assigned a somatic quality (SQ) score and marked as ‘weak_evidence’ if falling below a fixed threshold (SQ < 3.0 hard filtering). Variant calling results were saved in a VCF formatted file and a consensus sequence generated using the bcftools CONSENSUS command. Any base covered with less than 10 reads was assigned an N (hard-masking) and any variable base was assigned the major allele base by default. Leading and trailing masked bases were then removed from the consensus by default.

### Lineage and clade assignment

Consensus sequences were analyzed with the Pangolin and NextClade pipelines to generate the lineage and clade information, respectively. By default, the latest version of the PANGOLearn and NextClade databases were downloaded for the most up to date lineage and clade information.

### Data delivery to CDC

Data delivery to CDC was performed through our internal web application. Along with connecting to the BaseSpace file system, the application also connected to the CDC S3 data bucket using standard Amazon Web Services (AWS) S3 command line connection protocol using key based authentication [1]. Data transfer was performed on a per sample basis per CDC guidelines. For each sample the application created a file stack with pertinent files for samples that pass the quality thresholds. The application then opened a connection to the CDC S3 bucket and created a dated folder. Batch folders were created inside the dated primary folder when more than one batch of transfers were processed on a given day. One subfolder per sample was then created in the batch folder. The application then transferred all the pertinent files for the sample into the sample folder and continued this process until all sample data were transferred. The application created a cumulative metadata file containing the metadata associated with the samples transferred and transferred the file to the S3 bucket.

## Results

### Sample characteristics

A total of 24,441 SARS-CoV-2 positive samples (April to June of 2021) were sequenced (Additional file 1: Table S1). Of these, the majority (24,237) samples were SA, 131 and 73 were from NP and OP, respectively (Table 1). Of the 24, 441 individuals, 74% were from NJ and MN, with the remaining 26% distributed across the remaining 50 states. Where data was provided (21,125 patient samples) the mean age was 33 (ranging from 0 to 97) with a Sex distribution (20,868 patients) of 38% females, 46% males and 15% with no Sex data available (Table 1). Of the 24,441 positive individuals, ~ 60% self-identified with an ethnicity, ~ 10% Hispanic or Latino, ~ 0.02%, Black not of Hispanic Origin, ~ 50% Non-Hispanic or Latino and White not of Hispanic Origin ~ 0.13% and 14% were either other or unknown origin. The remaining declined to identify, or information was unavailable. Vaccination status was provided by 23,329 (~ 95%) of patients. Of those ~ 7% indicated yes while 88% indicated no, the remaining declined to provide status.

**Table 1:** Totals and percentages of samples sequenced and characteristics

Total samples	24,441
Sample type	
SA	24,237
NP	131
OP	73
Geographic location	
MN	16,485
NJ	1684
CA	386
Other U.S. states and territories	5886
Avg age years (range)	
Males	32.35 (0–94)
Females	32.17 (0–99)
Undisclosed	34.23 (6–74)
Mean	32.29 (0–99)
Sex (% samples sequenced)	
Male	11,418 (46.72)
Female	9450 (38.66)
Not identified	3573 (14.62)
Ethnicity (% samples sequenced)	
Hispanic or Latino	2518 (10.30)
Non-Hispanic or Latino	12,112 (49.56)
Others/unknown not disclosed	9811 (40.14)
Vaccination status (% yes/no/unknown)	
Yes	1757 (7.18)
No	21,572 (88.26)
Unknown	1113 (4.55)

### Genome coverage and ambiguity rates between sequencing instruments

**Fig. 2:**
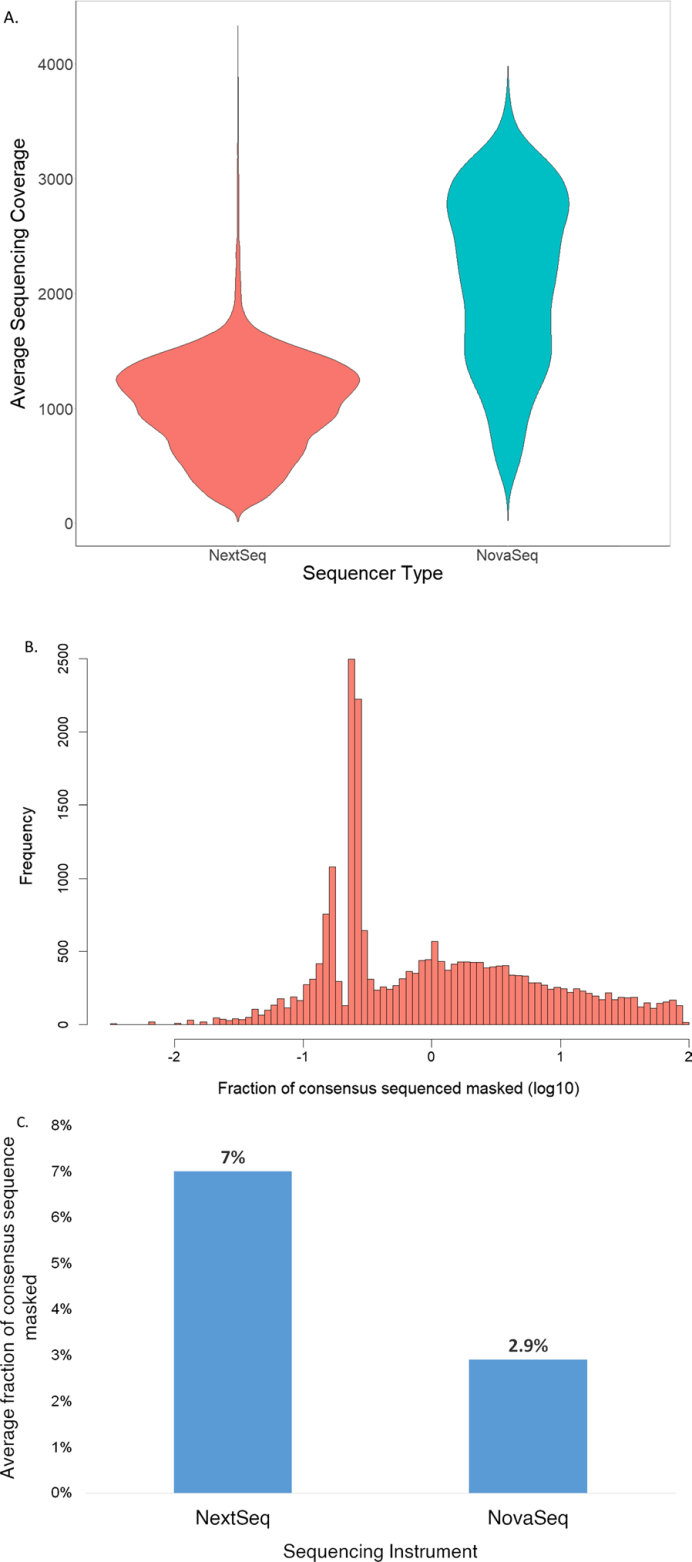
Genome coverage and ambiguity rates between sequencing instruments. **a** Distribution of average coverage over genome in samples sequenced using NextSeq550 and NovaSeq600. **b** Distribution of percent ambiguous nucleotides (masked) in consensus sequence. **c** Fraction of consensus sequence masked due to nucleotide ambiguity in samples sequenced on NextSeq550 and NovaSeq6000 instruments sequenced on NextSeq550 and NovaSeq600 instruments

Of all the samples, 73.5% (17,974) samples were sequenced on the NextSeq 550, while 26.46% (6467) were sequenced on the NovaSeq 6000. The average sequencing coverage between the two was ascertained using average sequencing coverage over the SARS-COV-2 genome and fraction of nucleotides masked due to sequencing ambiguity in the consensus sequence generated. The global average SARS-CoV-2 genome coverage for all samples sequenced across both instruments was 1324×. Samples run on the NovaSeq 6000 had twice the average coverage (2133×) compared to samples run on NextSeq550 (1034×) because more reads per sample were generated on the NovaSeq 6000. The fraction of masked nucleotides in consensus sequence generated was globally 5.9%. The ambiguity fraction rate using the NovaSeq6000 was 2.9% while genomes sequenced on the NextSeq550 had a higher ambiguity rate at 6.9% (Fig. 2a–c).

### Sequence data quality between sample collection methods

**Fig. 3:**
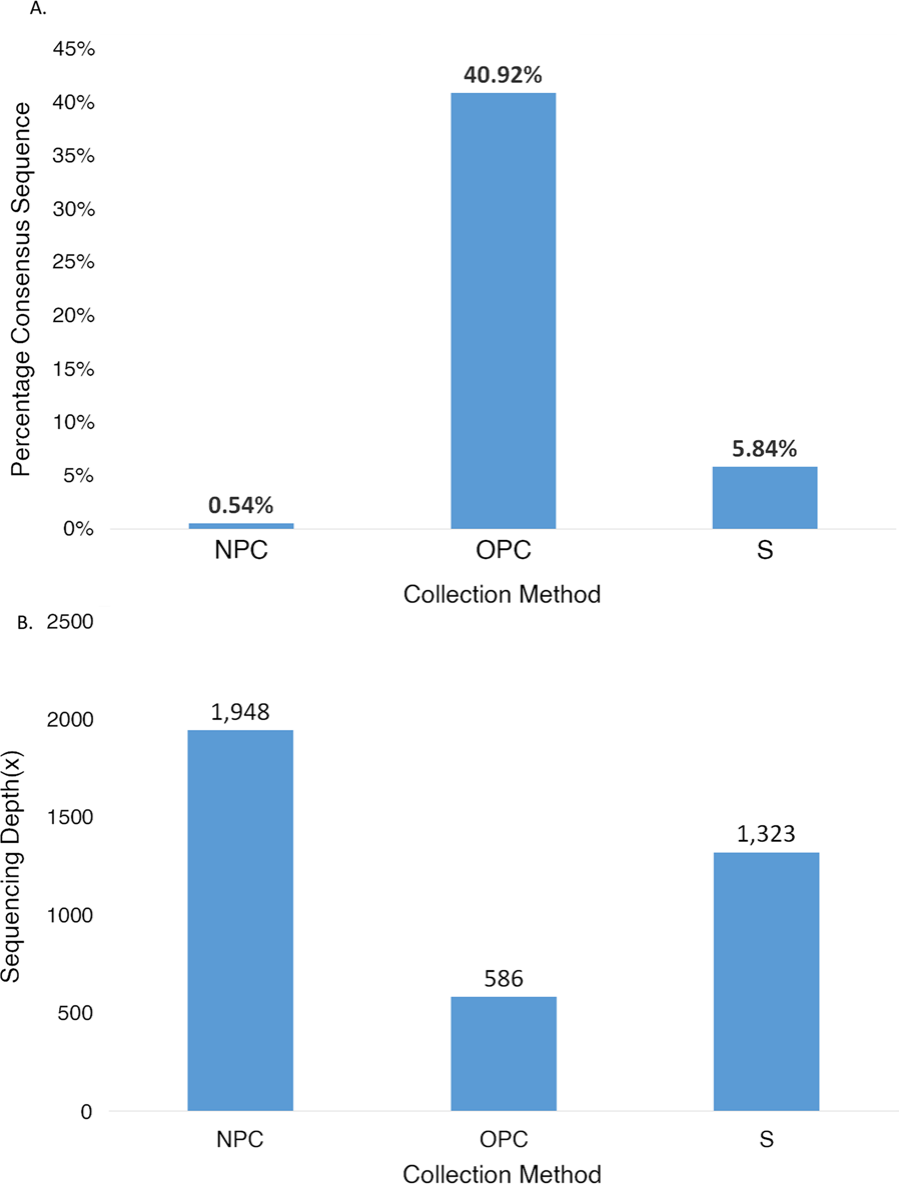
Sequence data quality between sample collection methods. **a** Average percentage of consensus sequence masked as ambiguous in the samples coming from three collection types (NP = Nasopharyngeal Collection, OP = Oropharyngeal collection, S = Saliva Collection). **b** Mean coverage (depth) of sequencing attained for samples coming from three collection types (NP = Nasopharyngeal Collection, OP = Oropharyngeal collection, S = Saliva Collection)

The mean RT-PCR Ct values for the three sample types used in this study across NP, OP and SA were 20.83, 21.83 and 22.70 respectively. By Tukey’s test, the mean Ct values differed significantly between SA and NP (p = 4e−7), but not between the other sample pairs. With respect to SARS-CoV-2 sequence data quality metrics, several sample specific patterns were identified. The rate of failure of analytical detection of SARS-CoV-2 sequence was highest in OP samples (4.11%, 3 of 73 samples), followed by SA samples (1.58%, 382 of 24,237 samples). The sample type exhibiting the lowest rate of SARS-CoV-2 detection failure was NP (0%, 0 of 131 samples). An important measure of input sample and sequence data quality is reflected in the percentage of consensus sequence masked as ambiguous. Higher values of this percentage are indicative of greater proportion of data that is not informative in variant determination. Figure 3a depicts the average percentage of consensus sequence masked as ambiguous for the three sample types used in this study. With a value of 0.5%, NP samples yielded the highest quality sequence data, while SA (5.84%) and OP swabs (41%) performing relatively less well. Another critical measure of quality, sequencing depth, was assessed for the three sample types as depicted in Fig. 3b. This analysis determined that NP and SA samples yielded the greatest depth, exhibiting values of 1948× and 1323×, respectively, while OP sample coverage was the lowest at 586×.

### Association between sequencing-based detection of SARS-CoV-2 virus and baseline RT-PCR Ct values

**Fig. 4:**
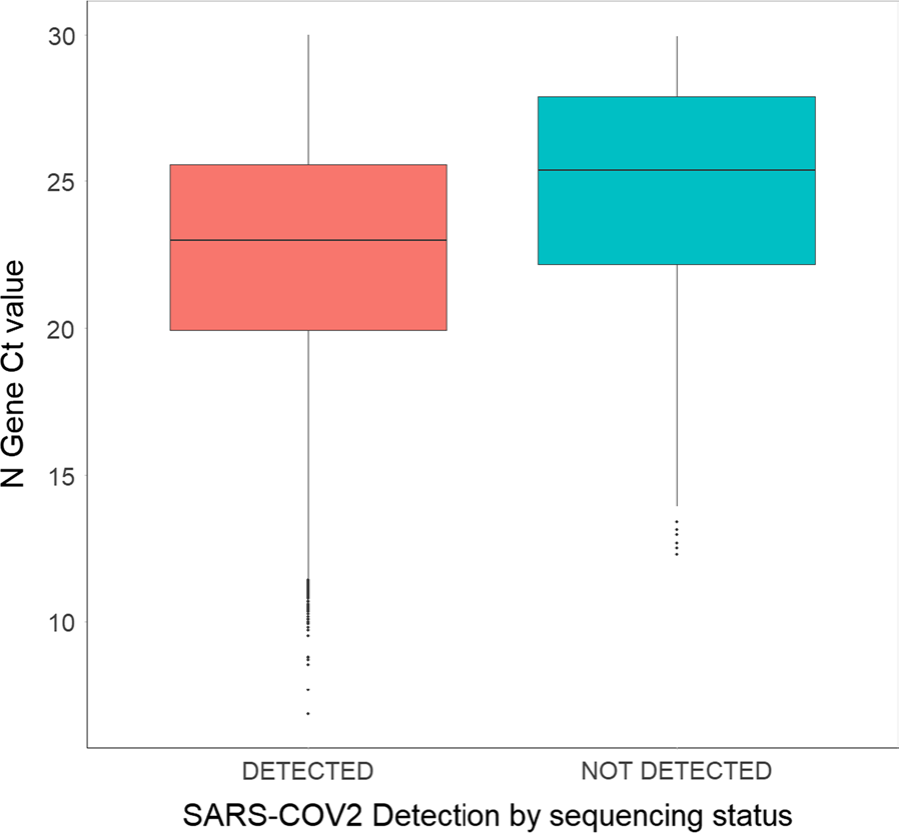
Association between sequencing-based detection of SARS-CoV-2 virus and baseline RT-PCR Ct Values. Correlation of detecting SARS-CoV-2 viral content in each sample tested with N gene RTPCR Ct values. A lower N gene Ct value was associated with enhanced detection of viral SARS-CoV-2 than those with a higher N gene Ct value as quantitated using RTPCR

Samples were selected for inclusion in the sequencing study based on a minimum threshold of SARS-CoV-2 detection by RT-PCR (N Gene Ct value threshold ≤ 30). Further investigation of the SARS-CoV-2 PCR data of the samples in this study reveals a direct correlation between the ability to detect SARS-COV-2 viral content by sequencing and the Ct values of the N gene. This observation held across all three sample types. Of the 24,441 samples sequenced, 385 (1.5%) were negative for SARS-CoV-2 virus by sequencing, while 24,056 samples were positive. Among the negative samples, the mean Ct value for the N Gene was 24.5, while for positive samples it was 22.5 (Fig. 4, Table 2).

**Table 2:** Mean Ct value ranges for three SARS-CoV-2 genes in detected and not detected samples

	# Samples	x̅ of N gene Ct	x̅ of S gene Ct	x̅ of ORF1Ab Ct
Not detected	385	24.56	24.75	24.79
Detected	24,056	22.57	22.47	22.64
Total	24,441	22.60	22.54	22.68

### Association between Ct value and genome coverage

**Fig. 5:**
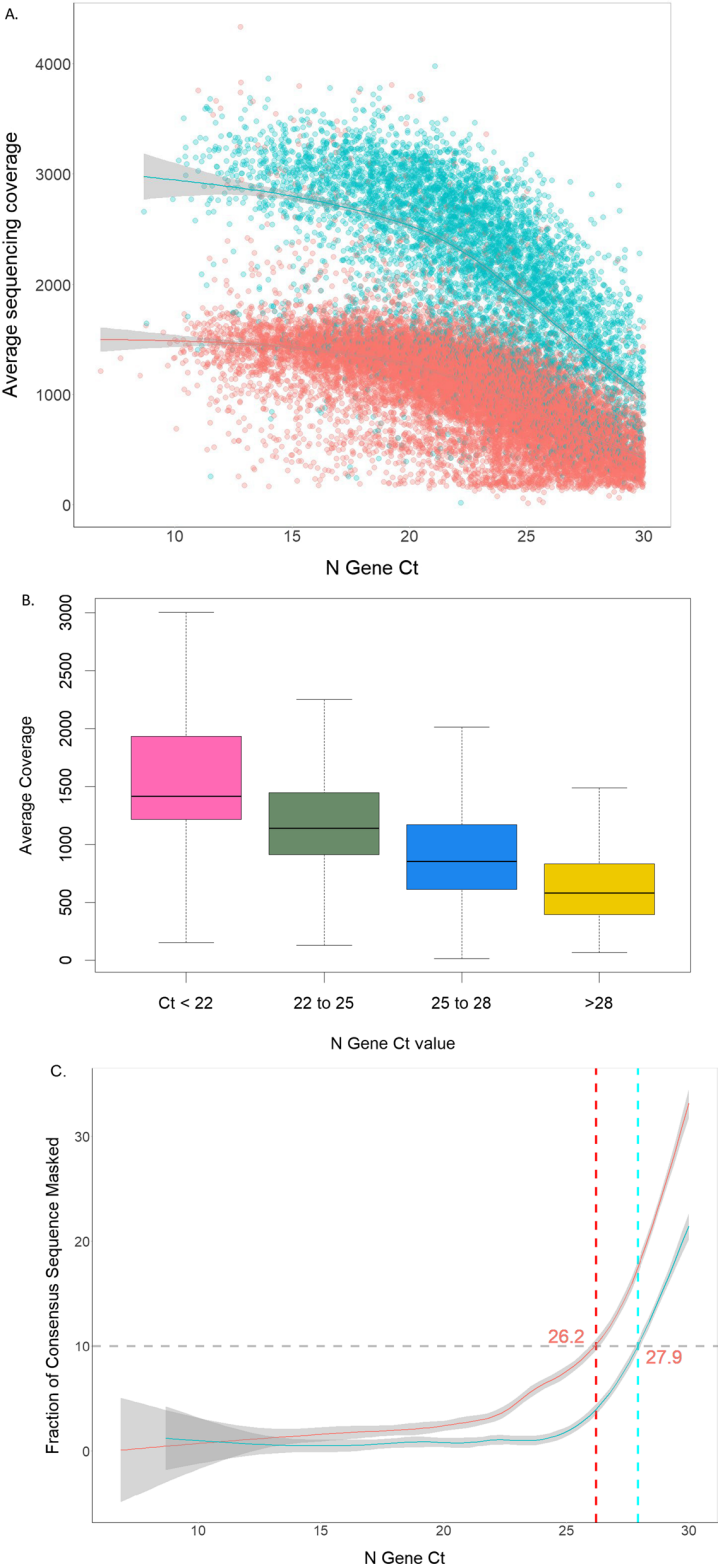
Association between Ct values and genome coverage. **a** Mean genome coverage of samples run on the two sequencing platforms is associated with baseline N gene Ct values of the samples. **b** Distribution of mean genome coverage achieved amongst samples from various Ct groups. **c** Ct values of 26.2 for NextSeq (red line) and 27.9 for NovaSeq (blue line) samples were identified as a selection resulting high quality (mean coverage > 100×, ambiguous nucleotide fraction in consensus sequence < 10%) sequences

We observed a strong inverse association between higher coverage and N gene Ct values as determined by RT-PCR (partial r, controlling for sequencer, = − 0.58, partial r-squared = 0.34, p < 1e−15) (Fig. 5) with higher Ct values resulting in lower overall mean genomic coverage. Spearman’s rank correlation coefficient between Mean Ct and % Genome Coverage was − 0.52 (p < 1e−15) for samples sequenced on the NovaSeq and − 0.75 (p < 1e−15) for samples sequenced on the NextSeq. To examine the relationship between baseline Ct value and mean genome coverage, we divided the samples into four groups, group 1 with Ct values < 22, group 2 with Ct values between 22 and 25, group 3 with Ct values between 25 and 28, and group 4 with Ct values > 28. We saw a clear difference in mean genome coverage among the four groups (Fig. 5). Group 1, with lowest Ct samples had the highest genomic coverage while group 4 with highest Ct values, had the lowest mean genomic coverage. We identified only eight samples in our dataset that had mean genome coverage less than 100×. Of the remaining 24,433 samples we determined that a mean Ct value of 26.5 for NextSeq 550 and 27.9 for NovaSeq 6000 was a threshold for producing high quality genome sequencing reads (Fig. 5a–c).

### Strain prevalence

**Fig. 6:**
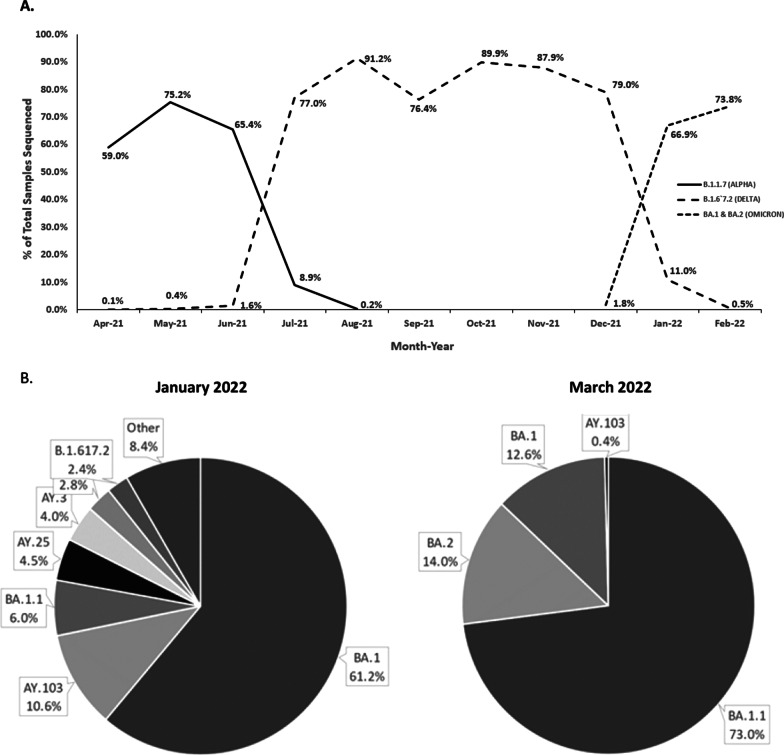
Trends in evolution of CDC designated variants of concern in our dataset from April 2021 to February 2022. **a** A total of 161 lineages of the SARS-CoV-2 variants were detected in the study from April 2021 to June 2021. The prevalence by percent total samples tested are indicated by each data point for the month through February 2022. The top 3 variants of concern by percent of total samples processed are depicted above. **b** Phylogenetic analysis shows the prevalence of variants as a % of positive samples sequenced in January of 2022 to March of 2022. Omicron lineage BA.16 in January 2022 accounted for ~ 61% of positive samples sequenced, where is in March of 2022 99% of all variants detected were lineages of Omicron (BA.1, BA.1.1, and BA.2)

We identified a total of 161 lineages of SARS-CoV-2 variants in our dataset of 24, 237 positive SARS-CoV-2 samples. The 10 most prevalent lineages are listed in Table 3. For a full list of all variants identified refer to Additional file 1: Table S1. The most prevalent lineage identified during April and June of 2021 was B.1.1.7 or the Alpha variant (65%, n = 15,806), followed by B.1.526 variant (5.54%, n = 1330). We identified 3 out of the 4 CDC variants of interest (B.1.525, B.1.526, B.1.617.1) and all CDC variants of concern B.1.1.7, B.1.351, B.1.351.2, B.1.351.3, B.1.617.2, P.1 and P.1 sublineages) in our study during this time period. The most prevalent variants detected were the Alpha variant between the months of and mid-June 2021, followed by a decline and a rapid increase in transmission of the Delta Variant from mid-June to the end of June 2021. The prevalence and transmission of Delta (B.1.617) continued to rise and was the most prevalent between the months of July and December 2021 (Fig. 6a). The only CDC variant of interest that was not identified in our study within the sample set and time frame tested was B.1.617.3. Further details on trends in lineage discovery in our study as the data continued to be accumulated between the months of June 2021 to February 2022 demonstrated a rapid rise and major prevalence of the Omicron variant BA.1 are provided in Additional file 1: Table S1 and Fig. 6a and b. Extending the data set into March 2022 (Fig. 6b) sub-lineages of BA.1, BA 1.1 and BA.2 continue to increase at the time of this submission.

**Table 3:** Incidence of CDC SARS-CoV-2 variants of concern in population of samples sequenced

Lineage	#Samples	% Of total sequenced
B.1.1.7	15,806	65.86
B.1.526	1330	5.54
B.1.429	1015	4.23
B.1.2	950	3.96
B.1.1.519	730	3.04
B.1.526.2	488	2.03
P.1	435	1.81
B.1.427	411	1.71
B.1	396	1.65

## Discussion

Genomics-based SARS-CoV-2 surveillance is an important tool for monitoring viral transmission during the current and future phases of the COVID-19 pandemic. In this work, we sequenced the genomes from > 24,000 SARS-CoV-2-positive samples collected during routine diagnostic testing. Furthermore, we analyzed the genomic epidemiology and sequencing applications of SARS-CoV-2 for the CDC surveillance program between March 2021 and June 2021, with a focus on variant and lineage determination, quality of sequencing results between sequencing instruments and the association between Ct values, genome coverage and variant detection.

Various NGS-based approaches have been developed to perform SARS-CoV-2 WGS using different sequencing platforms [12, 13]. These include direct RNA sequencing and metagenomics, amplicon-based methods and oligonucleotide capture-based methods. We employed COVIDseq for mass-scale SARS-CoV-2 genomic surveillance [14] and demonstrated that COVIDseq enables near-complete coverage of the SARS-CoV-2 genome. We used statistical analysis to demonstrate that there were differences in average coverage between the sequencing instruments with the NovaSeq 6000 having larger output per sample compared to the NextSeq 550. In addition, we determined that a Ct value of 26.5 for NextSeq and 27.9 for NovaSeq served as thresholds for producing high quality genomes, above these thresholds sequencing quality degraded. High quality genomes were identified as ones that have a fraction of ambiguous nucleotides in the consensus sequence of less than 10% and average genome coverage > 100×. Sequencing of samples with Ct values > 35 has previously been reported to show a sizable fraction of the reads are aligned to the human genome, independently of the method used to prepare the libraries, resulting in inconsistency in lineage and variant detections [14].

We demonstrate that reports regarding variants of concern, such as transmission of the Delta variant, are consistent with other US and international reports [15]. We identified a total of 161 lineages of the SARS-CoV-2 variants in our dataset, between April to June 2021, and the top 10 lineages were consistent with data reported to date beyond the present study. In addition, as is the nature of RNA viruses, each new variant and strains that are identified may gain a natural advantage and become the dominant strain [15, 16] (Fig. 6). Moreover, with the increased number of positive cases in summer and winter of 2021, these data may also be consistent with vaccine breakthrough infections [15]. However, additional studies are warranted. Additional variants, such as the Delta variant, where the variant of interest may not Although the Delta variant continues to represent most SARS-CoV-2 infections, sequencing data between the months of June and September 2021 are demonstrated increasing rates of transmission of another variant of concern, the Mu (B.2.621) variant, first identified in Colombia in January of 2021 [17]. Although the Mu variant accounts for only about 0.1% of cases within the present dataset, 77 positive samples with B.1.621 (Mu) have been identified since June of 2021.

By comparing the RT-PCR Ct values with the ability to detect variants of SARS-CoV2, a likely correlation with the clinical features of the infection may be inferred. Increased genome coverage associated with lower Ct values are likely due to higher viral load in each sample [18]. These results are consistent with previous reports where the ability to accurately detect variants correlates with lower Ct values [19]. However, most of these samples are from SA-based collections. Conflicting evidence regarding the sensitivity between various collection methods for detecting SARS-CoV-2 positive patients have been previously reported SARS-CoV-2 [6, 20, 21]. Thus comparison between SA and other collections method remains to be performed. The data presented in this study indicate that NP and SA are the optimal sample types for SARS-CoV-2 surveillance sequencing studies, both in terms of strain identification and sequencing depth of coverage. Additional data and studies will need to be performed to further elucidate sensitivities associated with collection methods. As demonstrated by the data above (Table 1), most samples tested derived from patients who indicated they were not vaccinated at the time of testing. Of those who were vaccinated, at the time of this study, the prominent variant in those breakthrough infections (April–June 2021) was the B.1.1.7 (UK) variant. During this same period 28 cases of the Delta (B.1.617) variant were identified. However, as demonstrated in Fig. 6a, from June to July 2021 the occurrence of the UK variant dramatically decreases with a significant rise in the Delta variant that also correlated with an increase is positive cases throughout the U.S. At that time this accounted for the prominent variant identified throughout the US. Vaccination status of samples sequenced from July through the remainder of 2021 are currently being collected (data not published). Thus, additional studies and analyses will be required to correlate, vaccination status, viral loads, sequencing variant impacts and clinical severity. The incomplete demographic data of all samples in aggregate which were sequenced limits the ability to stratify variant transmission between not only geographical regions but also within Sex, age and other factors that may contribute to increased transmission rate. In November of 2021, the more transmissible variant from South Africa B.1.1.529 (Omicron) was detected [22, 23]. This variant quickly became more transmissible and prevalent throughout not only the U.S but the world, accounting for 99% of all SARS-CoV-2 positive cases (Fig. 6a, b). Of interest is the concern that patients positive for this variant may be missed as the variant results in an S gene drop out. For these reasons it is imperative the appropriate methods for detection of positive SARS-CoV-2 patients can detect the N and Orf genes as well. At the time of this submission, sub-lineages of Omicron (BA.2) are increasing within the population accounting for increased positivity rates throughout Europe and is increasing within the U.S. [22] (Fig. 6b).

## Conclusion

In summary, the CDC surveillance screening program for SARS-CoV-2 variant transmission using whole viral genome sequencing is an important approach for population-based surveillance and control of viral transmission in the next phase of the COVID-19 pandemic. As SARS-CoV-2 strains continue to be sequenced by government, private, and academic entities all over the world, the sequencing data must continue to be shared publicly. These sequencing efforts will allow genomic-based surveillance of the virus and data sharing and contribute to efforts to understand population-level spread and control of the SARS-CoV-2 virus.

## Supplementary Information

**Additional file 1.** Sample accession numbers submitted and successfully retrieved from GISAID.

## Data Availability

High quality consensus sequences that resulted in definitive lineages as determined by CDC were successfully uploaded to GISAID (https://www.gisaid.org/). The accession numbers for each sample submitted to and approved by CDC by IBX are publicly available in GISAID database. See Additional file 1: Table S1 for the accession numbers retrieved from GISAID. Raw data are available upon reasonable request from the corresponding author.

## Abbreviations

RT-PCR: Real time polymerase chain reaction
CDC: Centers for Disease Control and Prevention
SARS-CoV-2: Severe acute respiratory syndrome coronavirus 2
COVID-19: Coronavirus disease 2019
U.S.: United States
IBX: Infinity-Biologix
SA: Saliva
NP: Nasopharyngeal
OP: Oropharyngeal
MN: Minnesota
NJ: New Jersey
N Gene: Nucleocapsid protein
Ct: Cycle threshold
RNA: Ribonucleic acid
EUA: Emergency Use Authorization
S gene: Coronavirus spike protein
ORF1ab: Open reading frames genes
NGS: Next generation sequencing
BSSH: Base Space Sequence Hub
BAM: Binary Alignment Map
SQ: Somatic quality
AWS: Amazon Web Services
